# Supplementary material for: Heparan sulfate proteoglycans present PCSK9 to the LDL receptor
Source: Nat Commun. 2017 Sep 11;8:503. doi: 10.1038/s41467-017-00568-7 (PMC5593881; doi:10.1038/s41467-017-00568-7)
Supplement: Supplementary file 1 — Supplementary Information [file 41467_2017_568_MOESM1_ESM.pdf]

Description of Supplementary Files

File name: Supplementary Information
Description: Supplementary figures, supplementary tables and supplementary references.

File name: Peer review file

Supplementary Figure 1

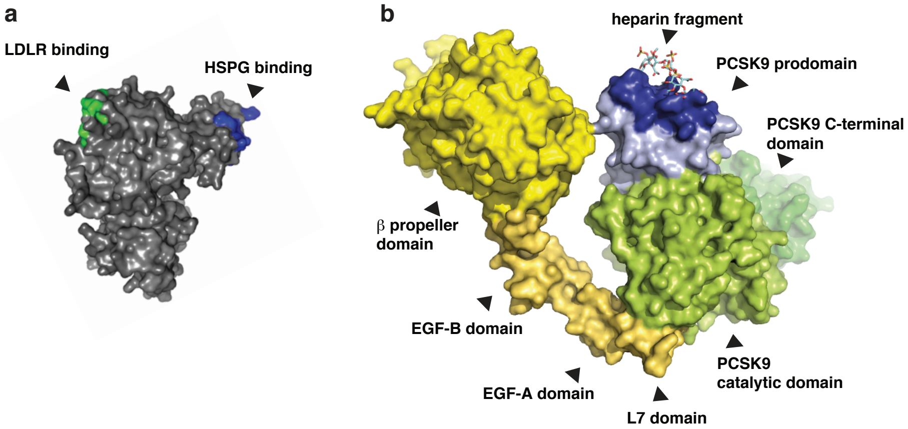

Supplementary Figure 1. PCSK9 HSPG binding domain
The HSPG binding domain is positioned opposite the LDLR binding site as shown in a space filling model of PCSK9 with the LDLR binding site highlighted in green and the HSPG binding site in blue (**a**) and further in a surface representation of PCSK9 in complex with a LDLR fragment (PDB ID: 3P5B) [1] (**b**). The LDLR fragment contains the beta propeller domain (yellow) and EGF domains A and B and L7 (orange), while PCSK9 C-terminal (green), catalytic domain (lime green), and prodomain (blue) is shown with the basic helix emphasized in dark blue. The modeled heparin fragment is shown as sticks.

Supplementary Figure 2

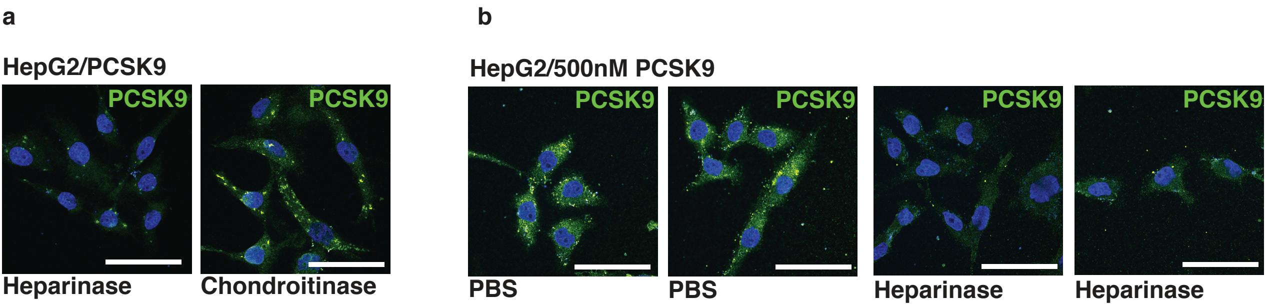

Supplementary Figure 2. Heparinase treatment abolish PCSK9 cell surface binding
(**a**) Immunofluorescence staining of non-permeabilized HepG2 expressing PCSK9 (green) after treatment with heparinase I or chondroitinase as indicated. (**b**) A different experiment in which HepG2 cells were treated with heparinase I or PBS (control), washed, and subsequently incubated with 500 nM PCSK9 (green) on ice where after the cells were fixed and stained in the absence of detergents to exclusively visualize surface localized PCSK9. Nuclei were stained with Hoechst (blue). Scale bars are 50 μm.

Supplementary Figure 3

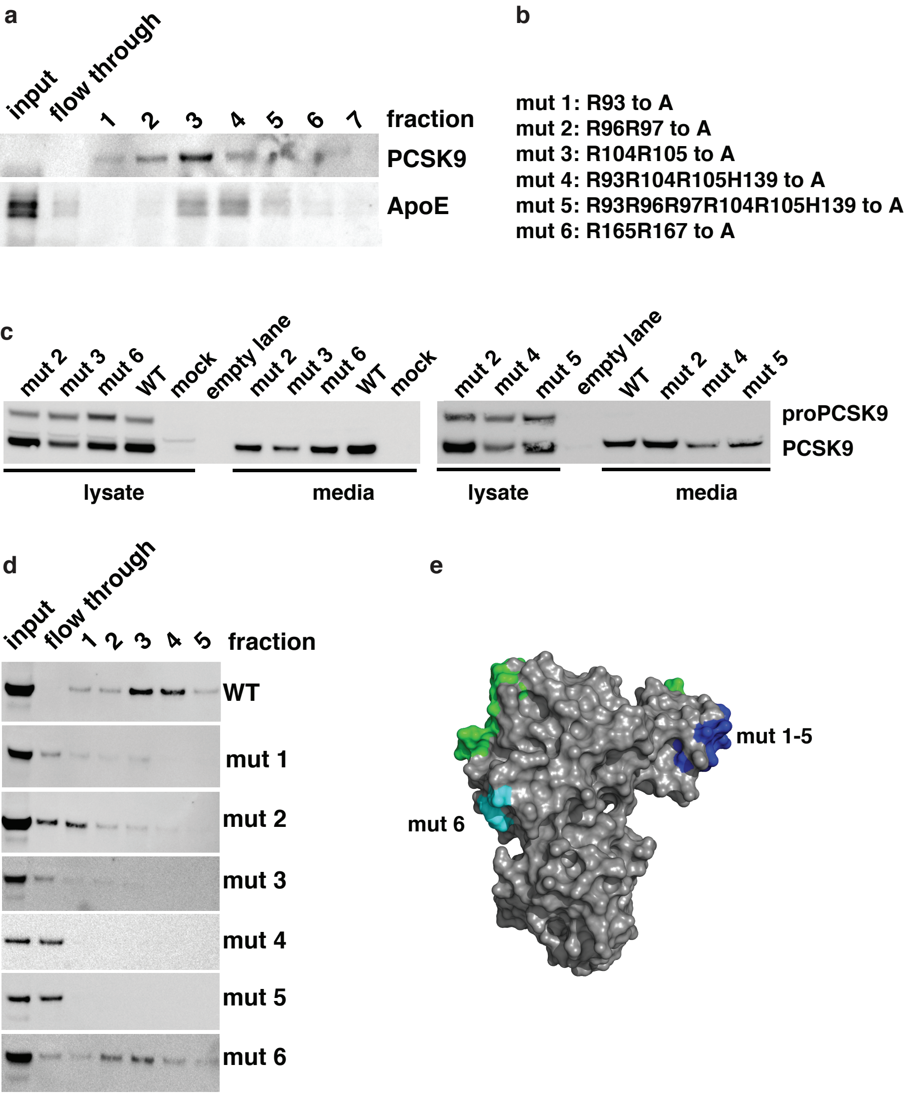

Supplementary Figure 3. PCSK9 is a heparin-binding protein

(**a**) Endogenous PCSK9 and ApoE binding to heparin was analyzed by affinity chromatography and WB. (**b**) List of PCSK9 mutants with combinations of basic residues substituted for alanine. (**c**) All PCSK9 mutants showed similar processing and secretion when expressed in CHO cells. Representative Western blots of cell lysate and conditioned media are shown. (**d**) All HSPG binding site mutants showed markedly reduced heparin binding, whereas mutation of two arginines outside the binding site (mut 6) has no major effect. (**e**) PCSK9 space filling model with the position of positively charged surface clusters containing amino acids changed in mut 1-5 (blue) and mut 6 (cyan), as well as LDLR binding domain (green), indicated.

Supplementary Figure 4

a

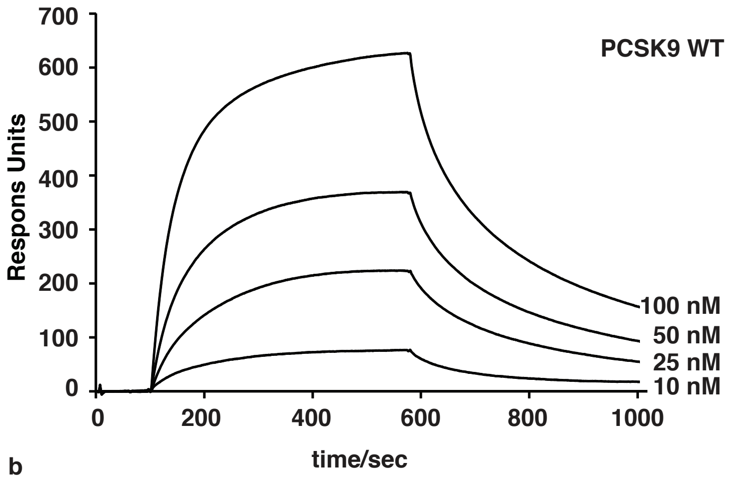

b

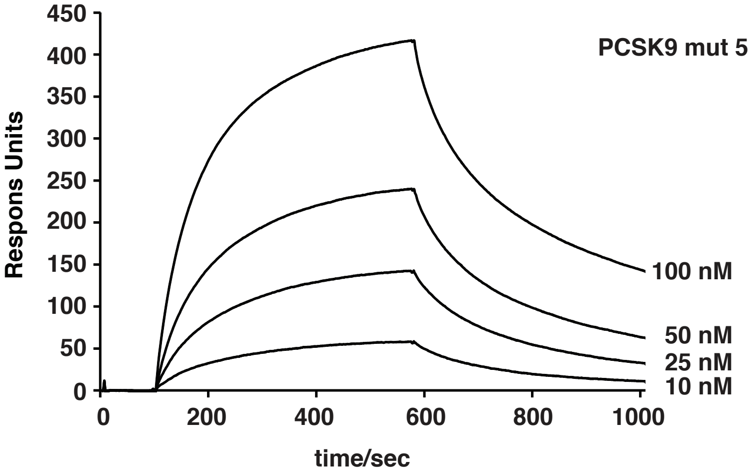

Supplementary Figure 4. Binding of PCSK9 to immobilized LDLR
Biacore sensorgrams showing that PCSK9 WT (**a**) and mut 5 (**b**) (10-100 nM) bind to the immobilized extracellular domain of LDLR with similar affinity estimated in the range (Kd=120-570 nM) as previously reported [2,3].

Supplementary Figure 5

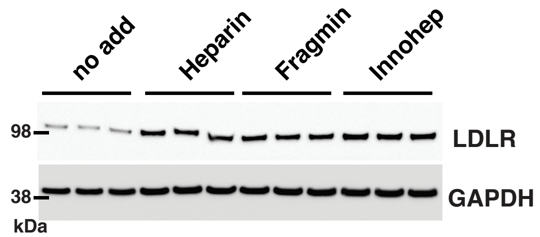

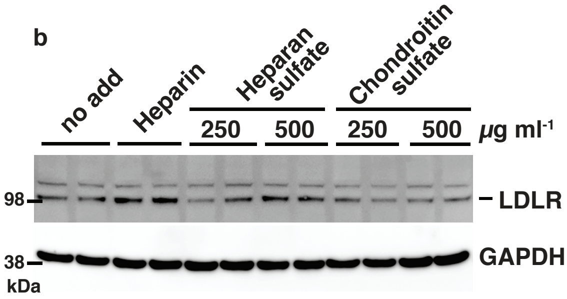

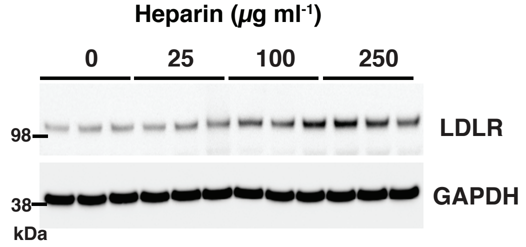

d

Ligand	K_D (μM)	Std. Error (μM)	R^2 (%)	n
Dextran sulfate 5000	180	1.30	92	3
Pentosan sulfate	381	1.26	95	2
Suramin	190	1.06	99	8
S-dC-36	4.8	1.6	94	4

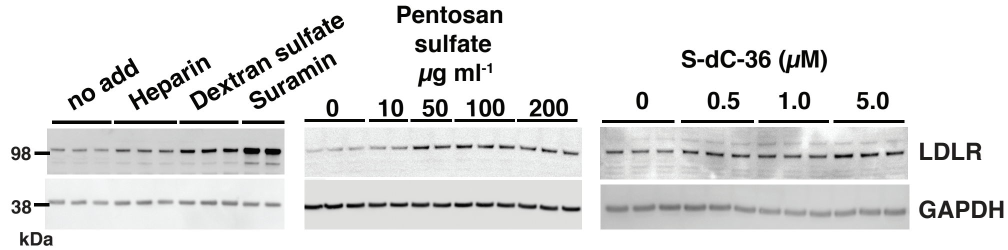

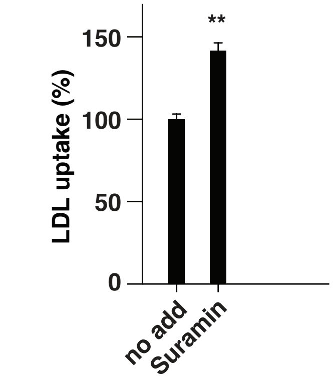

Supplementary Figure 5. Heparin and heparin mimetics are potent PCSK9 inhibitors
(**a**) Incubation with Fragmin (100 U/ml) or innohep (100 U/ml) resulted in a marked increase in cellular LDLR. (**b**) Incubation with heparan sulfate also increased LDLR levels, whereas chondroitin sulfate had no effect. (**c**) Dose dependent effect of heparin (0-25 U/ml) on LDLR levels in HepG2 cells (n=3). (**d**) KD values for the interaction between PCSK9 and heparin mimetics. (**e**) Representative Western blots showing LDLR levels in HepG2 cell lysate following incubation with the heparin mimetics dextran sulfate (200 μg/ml) and suramin (200 μg/ml), pentosan sulfate (0-200 μg/ml), and S-dC-36 (0-5.0 μM). GAPDH is used as loading control. (**f**) Incubation with suramin (200 μg/ml) increased HepG2 uptake of BODIPY-LDL (n=3). Statistical significance was evaluated using a two-tailed Student's t-test.

Supplementary Figure 6

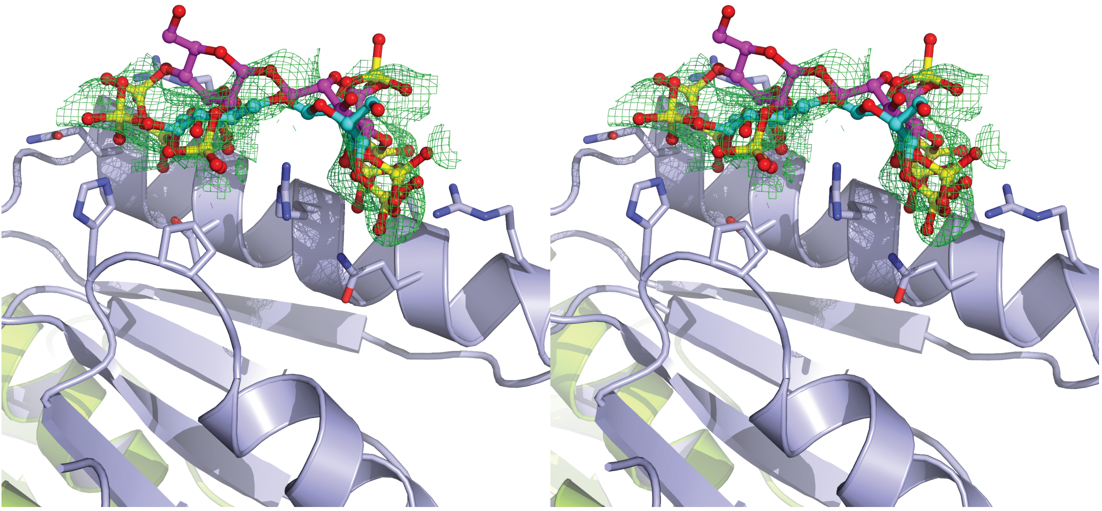

Supplementary Figure 6.
Wall-eyed stereo view of dextran sulfate and the corresponding Fo-Fc simulated annealing omit map countered at 2σ.

Supplementary Figure 7

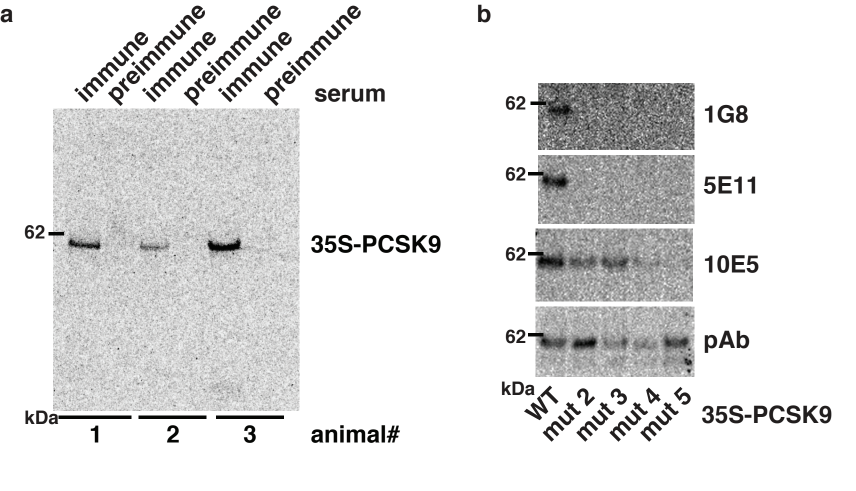

Supplementary Figure 7. Characterization of inhibitory mAbs directed at the PCSK9 HSPG binding site.

(**a**) Immunoprecipitation of radioactive-labelled PCSK9 using serum from three immunized rats. Preimmune serum is used as control. (**b**) The PCSK9 mutants listed in **Supplementary Fig. 3b** were used to determine if the amino acids predicted in Fig. 1a, b are involved in mAb binding. Clones 1G8 and 5E11 were found to depend on the presence of R96, R97, R104 and R105. (**c**) mAb 5E11 (5nM) does not inhibit binding of PCSK9 to LDLR in a PCSK9-(biotinylated)-LDLR binding assay, as control is used evolocumab (5nM). Statistical significance was evaluated using a two-tailed Student's t-test.

Supplementry Figure 8

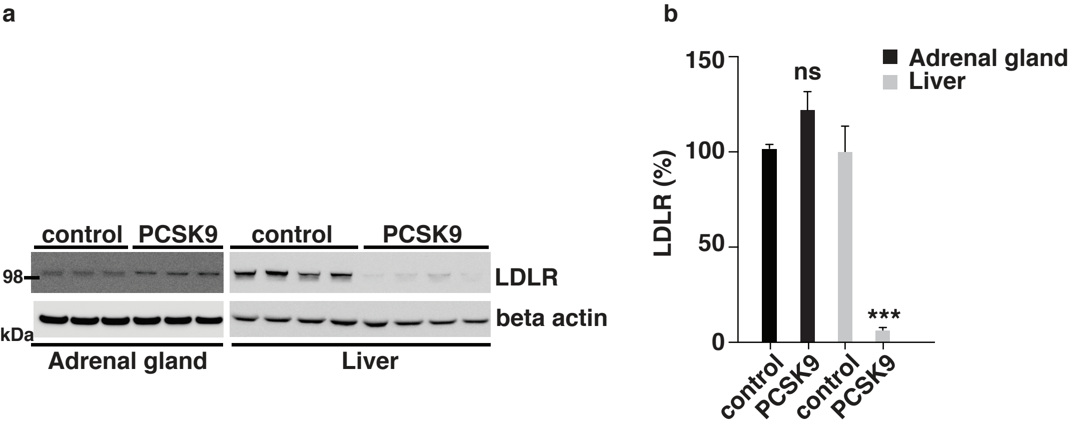

Supplementary Figure 8. PCSK9 specifically targets liver LDLR

(**a-b**) LDLR in adrenal gland (n=4, pooled tissue) of C57Bl/6j mice is unaffected by injection of 10 μg PCSK9 as assessed by WB of protein levels (quantification in **b**) in tissue 1 hour after injection. Liver LDLR levels from the same animals (n=4) are shown as control. Statistical significance was evaluated using a two-tailed Student's t-test.

Supplementary Figure 9

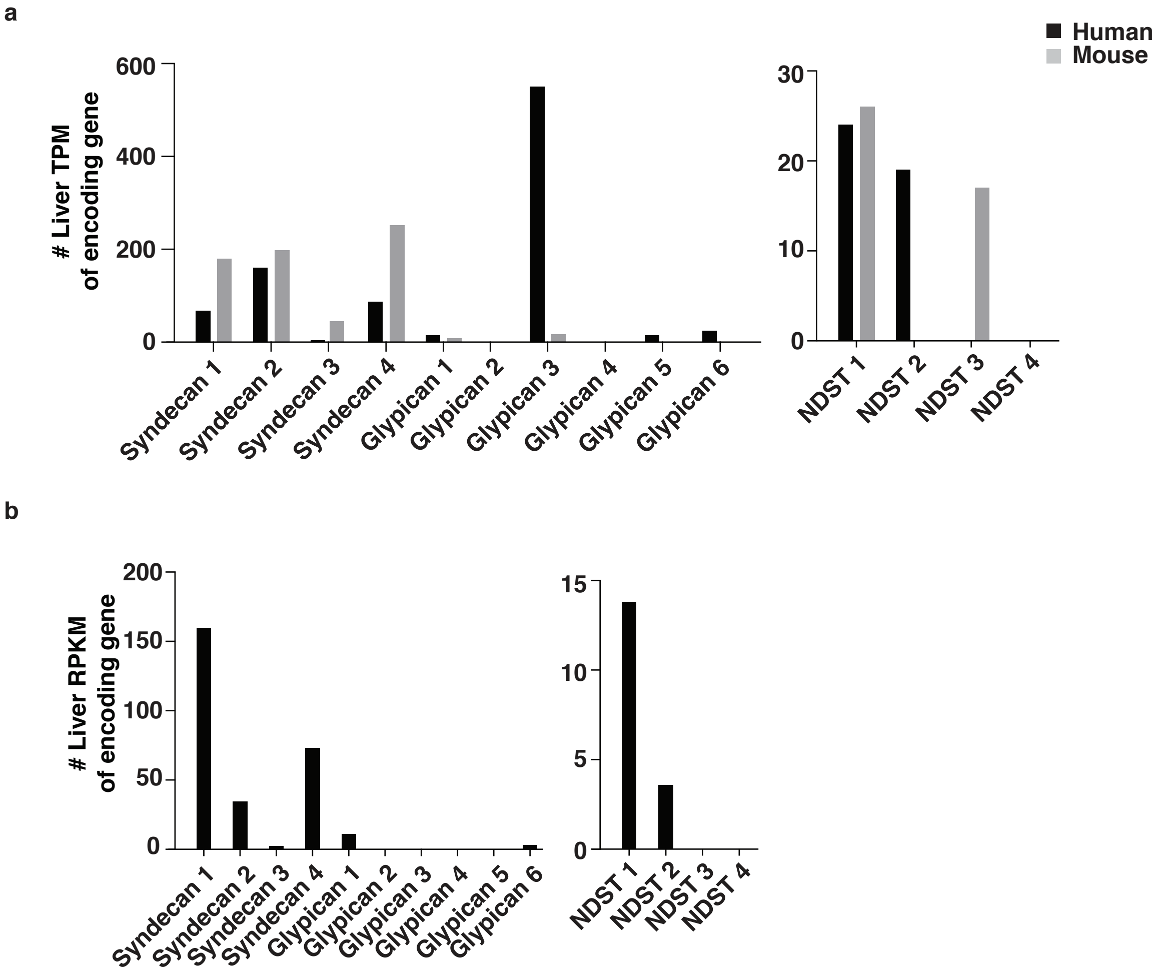

Supplementary Figure 9. Liver expression of syndecans, glypicans and NDSTs
(**a**) Data extracted from UniGene (The National Center of Biotechnology Information) showing numbers of hepatic transcripts per million transcripts (TPM) for the genes encoding syndecan 1-4, glypican 1-6 and NDST 1-4 in human and mouse. (**b**) Number of reads per kilobase per million mapped reads (RPKM) of the same genes in human liver as listed in the GTEx Portal (The Broad Institute of MIT and Harvard).

Supplementary Figure 10

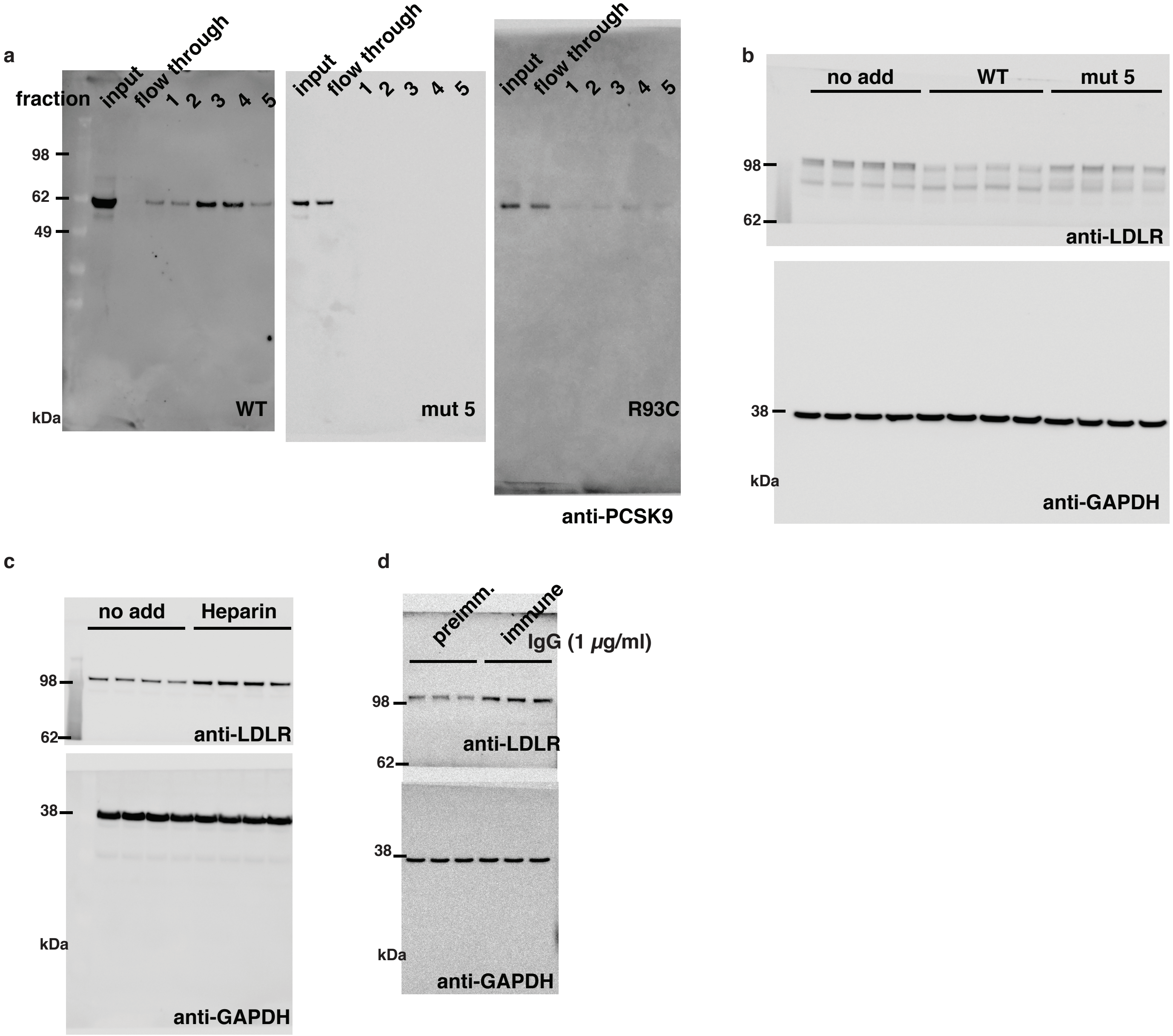

Supplementary Figure 10. Uncropped Images of Western blots from Figures 1-5
Uncropped Western blots from Fig. 1e (**a**), Fig. 1f (**b**), Fig. 1i (**c**), Fig. 5c (**d**).

Supplementary Figure 11

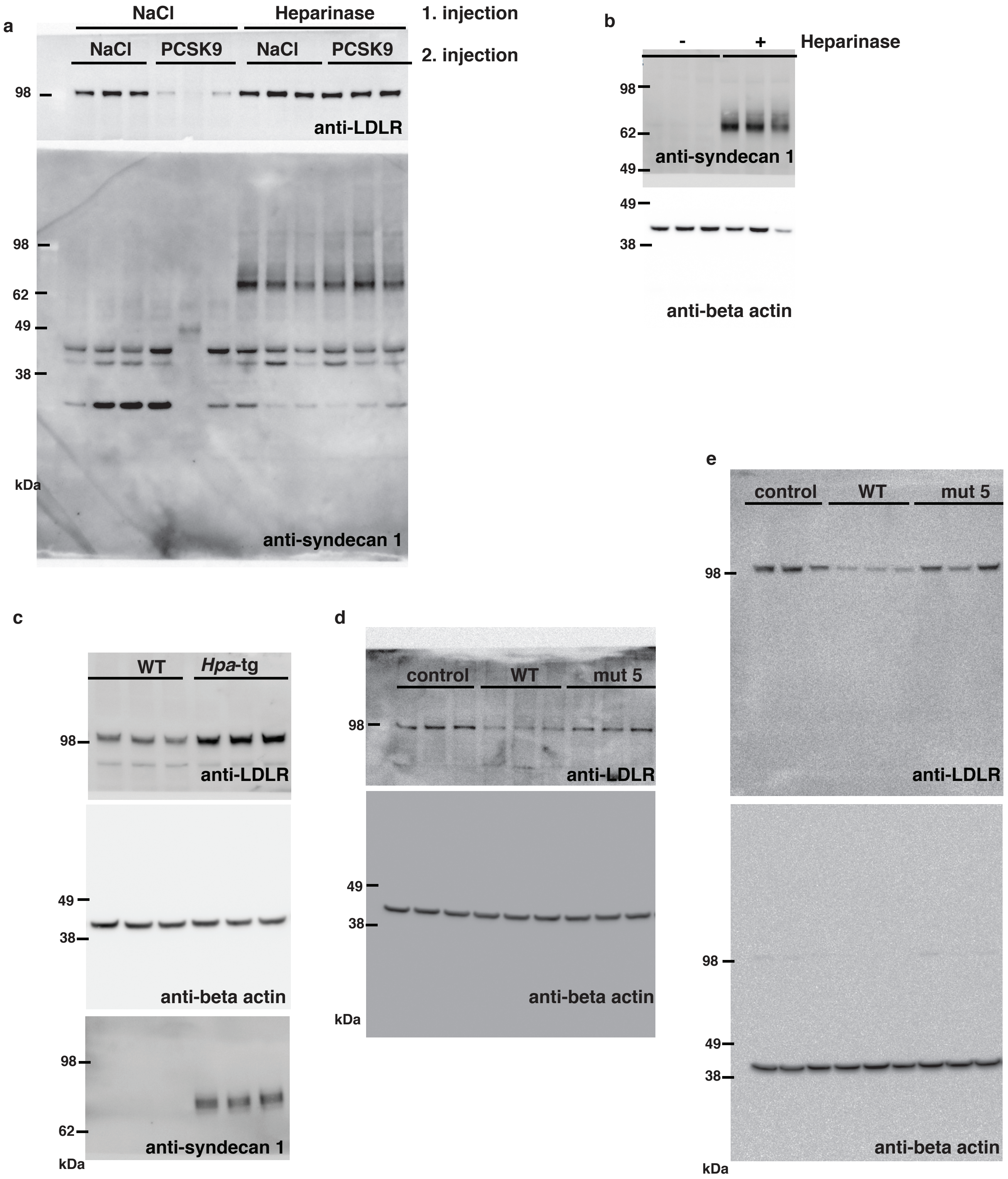

Supplementary Figure 11. Uncropped Images of Western blots from Figures 6-7
Uncropped Western blots from Fig. 6a (**a**), Fig. 6d (**b**),Fig. 6f (**c**), Fig. 7d (**d**), Fig. 7f (**e**).

Supplementary Table 1. Crystal structure refinement statistics

	3H42	3H42 + refinement	3H42 + refined Dextran Sulfate	Final refined model
**Rwork/Rfree**	0.191/0.209	0.176/0.200	0.175/0.198	0.167/0.195
**No of. Atoms**				
**Protein**	7630	7627	7627	7624
**Ion**	1	1	1	2
**Water**	568	568	568	788
**Ligand**			86	86
**B-factors**				
**Protein**	46.23	46.32	46.02	47.32
**Ion**	62.63	53.19	54.18	53.45
**Water**	50.04	47.9	47.72	52.08
**Ligand**			85.9	86.9
**R.m.s deviations**				
**Bond lengths [Å]**	0.005	0.008	0.009	0.009
**Bond angles [^{o}]**	0.852	0.89	0.95	0.96
**Ramachandran statistics**				
**Favored [%]**	97.4	97.4	97.6	96.3
**Allowed [%]**	2.6	2.5	2.3	3.49
**Outlier [%]**	0	0.1	0.1	0.2
**Rotamer outliers [%]**	2.28	0.36	0.36	0.36

Supplementary Table 2. Synthetic extracellular matrix glycans.

Code	Name	Trivial name	Reference
**1**	GlcN-6,N-disulfate(a1-4)IdoA-2-sulfate(a1-4)GlcN-6,N-disulfate(a1-4)IdoA-2-sulfate(a1-4)GlcN-6,N-disulfate(a1-4)IdoA-2-sulfate(a1-1)AminoLinker1	Heparin I	[4,5]
**2**	GlcNAc-6-sulfate(a1-4)IdoA-2-sulfate(a1-4)GlcNAc-6-sulfate(a1-4)IdoA-2-sulfate(a1-4)GlcNAc-6-sulfate(a1-4)IdoA-2-sulfate(a1-1)aminopentanol	Heparin II	[4,5]
**3**	GlcN-N-sulfate(a1-4)IdoA-6-sulfate(a1-4)GlcN-N-sulfate(a1-4)IdoA-6-sulfate(a1-4)GlcN-N-sulfate(a1-4)IdoA-6-sulfate(a1-1)aminopentanol	Heparin III	[4,5]
**4**	GlcNAc(a1-4)IdoA(a1-4)GlcNAc(a1-4)IdoA(a1-4)GlcNAc(a1-4)IdoA(a1-1)aminopentanol	Heparin IV	[4,5]
**5**	IdoA-2-sulfate(a1-4)GlcN-N-sulfate(a1-4)IdoA-2-sulfate(a1-4)GlcN-N-sulfate(a1-4)IdoA-2-sulfate(a1-4)GlcN-N-sulfate(a1-1)aminopentanol	Heparin V	[4,5]
**6**	IdoA-2-sulfate(a1-4)GlcNAc(a1-4)IdoA-2-sulfate(a1-4)GlcNAc(a1-4)IdoA-2-sulfate(a1-4)GlcNAc(a1-1)aminopentanol	Heparin VI	[4,5]
**7**	GlcN-6,N-disulfate(a1-4)IdoA-2-sulfate(a1-4)GlcN-6,N-disulfate(a1-4)IdoA-2-sulfate(a1-1)AminoLinker1	Heparin VII	[4,5]
**8**	IdoA(a1-4)GlcN-N-sulfate(a1-4)IdoA(a1-4)GlcN-N-sulfate(a1-1)aminopentanol	Heparin VIII	[4,5]
**9**	IdoA(a1-4)GlcN(a1-4)GlcA(b1-3)GlcNAc(a1-1)AminoLinker1	Heparin IX	[6,7]
**10**	GlcN-6,N-disulfate(a1-4)IdoA-2-sulfate(a1-1)AminoLinker1	Heparin X	[4,5]
**11**	Ido-2,4-disulfate(a1-1)AminoLinker1	Heparin XI	[4,5]
**12**	GlcN-6,N-disulfate(a1-1)AminoLinker1	Heparin XII	[4,5]
**13**	IdoA(a1-1)AminoLinker1	Heparin XIII	[4,5]
**14**	Natural heparin		
**15**	Neu5Ac(a2-6)Gal(b1-4)GlcNAc(b1-3)Gal(b1-4)Glc(b1-1)aminohexanol	Neu5Aca(2-6)Galb(1-4)penta	[8]
**16**	Neu5Ac(a2-3)Gal(b1-4)GlcNAc(b1-3)Gal(b1-4)Glc(b1-	Neu5Aca(2-3)Galb(1-3)penta	[8]

	1)aminohexanol		
**17**	Fuc(a1-3)[Neu5Ac(a2-3)Gal(b1-4)]GlcNAc(b1-3)Gal(b1-4)Glc(b1-1)aminohexanol	Sialyl LewisX	[9]
**18**	Neu5Ac(a2-6)Gal(b1-4)Glc(b1-1)aminohexanol	Neu5Aca(2-6)Gal GM3	[8]
**19**	Neu5Ac(a2-3)Gal(b1-4)Glc(b1-1)aminohexanol	Neu5Aca(2-3)Gal GM3	[8]
**20**	Neu5Ac(a2-6)Gal(b1-4)GlcNAc-6-sulfate(b1-1)aminohexanol	Neu5Aca(2-6)GalSO3H	
**21**	Neu5Ac(a2-6)GalNAc(a1-1)aminopentanol	Sialyl-Tn (STn) Antigen	
**22**	Neu5Ac(a2-8)Neu5Ac(a2-3)[GalNAc(b1-4)]Gal(b1-4)Glc(b1-1)aminopentanol	GD2 (Aminolinker)	
**23**	Neu5Ac(a2-3)Gal(b1-4)Glc(b1-1)aminopentanol	GM3 (Aminolinker)	[10]
**24**	GalNAc-4-sulfate(b1-1)aminopentanol	Dermatan GalNAc monosaccharide, 6;sulfated	[11]
**25**	IdoA-252,4-disulfate(a1-1)aminopentanol	Iduronic acid disulfate monosaccharide	[12]
**26**	IdoA(a1-3)GalNAc-4-sulfate(b1-1)aminopentanol	Dermatan monosulfated disaccharide	[11]
**27**	IdoA-2-sulfate(a1-3)GalNAc-4-sulfate(b1-1)aminopentanol	Dermatan disulfated disaccharide	[11]
**28**	IdoA(a1-3)GlcNAc(b1-1)aminopentanol	Dermatan nonsulfated disacchidare	[11]
**29**	Gal(b1-4)GlcNAc(b1-3)Gal(b1-4)GlcNAc(b1-1)aminopentanol	LacNAc repeat dimer	
**30**	Gal(b1-4)GlcNAc(b1-3)[Gal(b1-4)GlcNAc(b1-6)]Gal(b1-4)GlcNAc(b1-1)aminopentanol	LacNAc repeat branched trimer	
**31**	Gal(b1-4)GlcNAc(b1-3)Gal(b1-4)GlcNAc(b1-3)Gal(b1-4)GlcNAc(b1-1)aminopentanol	LacNAc repeat linear trimer	
**32**	Gal-6-sulfate(b1-4)GlcNAc(b1-3)Gal-6-sulfate(b1-4)GlcNAc(b1-1)aminopentanol	Keratan sulfate 2 repeating units	
**33**	Gal-3,6-disulfate(b1-4)GlcNAc(b1-3)Gal-6-sulfate(b1-4)GlcNAc(b1-1)aminopentanol	Keratan sulfate 2 repeating units	
**34**	Gal(b1-4)GlcNAc-6-sulfate(b1-3)Gal(b1-4)GlcNAc-6-sulfate(b1-1)aminopentanol	Keratan sulfate 2 repeating units	
**35**	Gal-6-sulfate(b1-4)GlcNAc-6-sulfate(b1-3)Gal-6-sulfate(b1-4)GlcNAc-6-sulfate(b1-1)aminopentanol	Keratan sulfate 2 repeating units	

SUPPLEMENTARY REFERENCES

1. Lo Surdo, P. et al. Mechanistic implications for LDL receptor degradation from the PCSK9/LDLR structure at neutral pH. *EMBO Rep* **12**, 1300-5 (2011).
2. Fisher, T.S. et al. Effects of pH and low density lipoprotein (LDL) on PCSK9-dependent LDL receptor regulation. *J Biol Chem* **282**, 20502-12 (2007).
3. Cunningham, D. et al. Structural and biophysical studies of PCSK9 and its mutants linked to familial hypercholesterolemia. *Nat Struct Mol Biol* **14**, 413-9 (2007).
4. Noti, C., de Paz, J.L., Polito, L. & Seeberger, P.H. Preparation and use of microarrays containing synthetic heparin oligosaccharides for the rapid analysis of heparin-protein interactions. *Chemistry* **12**, 8664-86 (2006).
5. de Paz, J.L., Spillmann, D. & Seeberger, P.H. Microarrays of heparin oligosaccharides obtained by nitrous acid depolymerization of isolated heparin. *Chem Commun (Camb)*, 3116-8 (2006).
6. Hecht, M.L. et al. Natural cytotoxicity receptors NKp30, NKp44 and NKp46 bind to different heparan sulfate/heparin sequences. *J Proteome Res* **8**, 712-20 (2009).
7. Adibekian, A. et al. De novo synthesis of uronic acid building blocks for assembly of heparin oligosaccharides. *Chemistry* **13**, 4510-22 (2007).
8. Hanashima, S. & Seeberger, P.H. Total synthesis of sialylated glycans related to avian and human influenza virus infection. *Chem Asian J* **2**, 1447-59 (2007).
9. Hanashima, S., Castagner, B., Esposito, D., Nokami, T. & Seeberger, P.H. Synthesis of a sialic acid alpha(2-3) galactose building block and its use in a linear synthesis of sialyl Lewis X. *Org Lett* **9**, 1777-9 (2007).
10. Fair, R.J., Hahm, H.S. & Seeberger, P.H. Combination of automated solid-phase and enzymatic oligosaccharide synthesis provides access to alpha(2,3)-sialylated glycans. *Chem Commun (Camb)* **51**, 6183-5 (2015).
11. Kandasamy, J., Schuhmacher, F., Hahm, H.S., Klein, J.C. & Seeberger, P.H. Modular automated solid phase synthesis of dermatan sulfate oligosaccharides. *Chem Commun (Camb)* **50**, 1875-7 (2014).
12. Nonaka, M. et al. Synthetic di-sulfated iduronic acid attenuates asthmatic response by blocking T-cell recruitment to inflammatory sites. *Proc Natl Acad Sci U S A* **111**, 8173-8 (2014).
